# Spatial and Temporal Analysis of *Plasmodium knowlesi* Infection in Peninsular Malaysia, 2011 to 2018

**DOI:** 10.3390/ijerph17249271

**Published:** 2020-12-11

**Authors:** Wei Kit Phang, Mohd Hafizi Abdul Hamid, Jenarun Jelip, Rose Nani Mudin, Ting-Wu Chuang, Yee Ling Lau, Mun Yik Fong

**Affiliations:** 1 Department of Parasitology, Faculty of Medicine, University of Malaya, Kuala Lumpur 50603, Malaysia; weikitphang@gmail.com (W.K.P.); lauyeeling@um.edu.my (Y.L.L.); fongmy@um.edu.my (M.Y.F.); 2 Disease Control Division, Ministry of Health Malaysia, Putrajaya 62000, Malaysia; dr.mhafizi@moh.gov.my (M.H.A.H.); jenarun@moh.gov.my (J.J.); drrose@moh.gov.my (R.N.M.); 3 Department of Molecular Parasitology and Tropical Diseases, School of Medicine, College of Medicine, Taipei Medical University, Taipei 11031, Taiwan

**Keywords:** spatial analysis, time series analysis, demography, malaria, *Plasmodium knowlesi*, Peninsular Malaysia

## Abstract

The life-threatening zoonotic malaria cases caused by *Plasmodium knowlesi* in Malaysia has recently been reported to be the highest among all malaria cases; however, previous studies have mainly focused on the transmission of *P. knowlesi* in Malaysian Borneo (East Malaysia). This study aimed to describe the transmission patterns of *P. knowlesi* infection in Peninsular Malaysia (West Malaysia). The spatial distribution of *P. knowlesi* was mapped across Peninsular Malaysia using Geographic Information System techniques. Local indicators of spatial associations were used to evaluate spatial patterns of *P. knowlesi* incidence. Seasonal autoregressive integrated moving average models were utilized to analyze the monthly incidence of knowlesi malaria in the hotspot region from 2012 to 2017 and to forecast subsequent incidence in 2018. Spatial analysis revealed that hotspots were clustered in the central-northern region of Peninsular Malaysia. Time series analysis revealed the strong seasonality of transmission from January to March. This study provides fundamental information on the spatial distribution and temporal dynamic of *P. knowlesi* in Peninsular Malaysia from 2011 to 2018. Current control policy should consider different strategies to prevent the transmission of both human and zoonotic malaria, particularly in the hotspot region, to ensure a successful elimination of malaria in the future.

## 1. Introduction

Over the decades, malaria has persisted as one of the major vector-borne parasitic diseases globally. Its impacts are geographically variable, depending on the intensity of transmission and the parasite species involved [1]. According to the World Health Organization report, an estimated 228 million malaria cases were reported in at least 80 countries and territories in 2018 [2]. With the effort of the roll back malaria initiative, there has been a significant progress in reducing malaria morbidity and mortality through strategies such as comprehensive disease surveillance, distribution of insecticide-treated bednets and the use of highly effective artemisinin combination medications [2].

In Malaysia, the Malaria Eradication Program was introduced in 1967, which continued until the 80s. The program has resulted in a significant reduction in malaria cases by approximately 94.3% in Peninsular Malaysia [3]. The incidence of human malaria from *Plasmodium vivax* and *Plasmodium falciparum* have dropped steadily nationwide. However, the emergence of zoonotic malaria caused by *Plasmodium knowlesi* has become the main cause of clinical malaria in Malaysia [1]. This has certainly challenged Malaysia’s efforts to eliminate malaria.

*P. knowlesi* is a simian malaria parasite which is prevalent in Southeast Asia and gaining prominence for its role in the increasing human malaria cases [4]. Its clinical manifestations typically include fever, chills, headache, malaise, rigors, and anorexia [5]. Severe cases can be highly life-threatening. A recent study reported that the average case fatality rate of *P. knowlesi* infection in Peninsular Malaysia from 2013 to 2017 was 1.2% [6]. It is often misdiagnosed via microscopy as P. malariae infection owing to morphological similarities between the two species at the trophozoite, schizont, and gametocyte stages [7,8]. This limitation warrants the need of molecular diagnostic techniques such as polymerase chain reaction (PCR), to identify and distinguish *P. knowlesi* as well as other *Plasmodium* species infections by amplification of species–specific gene targets. *P. knowlesi* is mainly transmitted to human from non-human primates via bite of forest-dwelling *Anopheles* mosquitoes from the Leucosphyrus group [9]. However, the possibility of human-to-human transmission has been proposed in a previous entomological study in Vietnam [10].

Malaria case detection in Peninsular Malaysia utilizes three systems: active case detection (ACD), mass blood survey (MBS), and passive case detection (PCD). ACD involves screening for febrile individuals in localities experiencing outbreaks or within high-risk groups such as the military, indigenous people, rural settlers, and migrant workers. MBS requires at least 80% of the community members who receive insecticide-treated nets (ITNs) and residual spraying to be tested. Moreover, MBS is applied as a response during outbreaks when health officers are required to screen every individual within an outbreak locality. PCD consists of detecting malaria cases among patients visiting health centers for their treatment. Most confirmed human and simian malaria cases were detected through the PCD approach.

Understanding the spatial and temporal patterns of *P. knowlesi* infection is important not only for case detection but also for resource allocation. Spatial analysis is commonly used for disease hotspot identification and evaluation of patterns of epidemics. Several studies have used spatial-temporal analysis to detect the clustering of malaria cases [11,12,13]. A better understanding of the spatial distribution of knowlesi malaria is useful to improve intervention programs and health resource allocation. In addition, cluster detection can aid in more focused epidemiological surveillance of macaque reservoir hosts and research on the bionomics of mosquito vectors. Studies based on spatial-temporal analysis of malaria distribution in Sabah, Malaysian Borneo (East Malaysia) have been conducted extensively [14,15,16]. However, a similar study investigating transmission patterns in Peninsular Malaysia (West Malaysia) is lacking. Therefore, this study aimed to use spatial-temporal analysis to identify *P. knowlesi* infection hotspots in Peninsular Malaysia at the district level between 2011 and 2018. Furthermore, state-wide demographic parameters of knowlesi malaria in Peninsular Malaysia were also investigated to reveal fundamental epidemiological characteristics of *P. knowlesi* infections.

## 2. Materials and Methods

### 2.1. Geography and Demography of Study Areas

Malaysia is a country in Southeast Asia, and it is separated by the South China Sea into two regions, Peninsular Malaysia and Malaysian Borneo. The national census in 2010 estimated the total population of Malaysia at 28.3 million [17]. The annual citizen population growth rate was projected at 1.6% in 2010. However, the growth rate declined to 1.1% in 2018.

**Figure 1:**
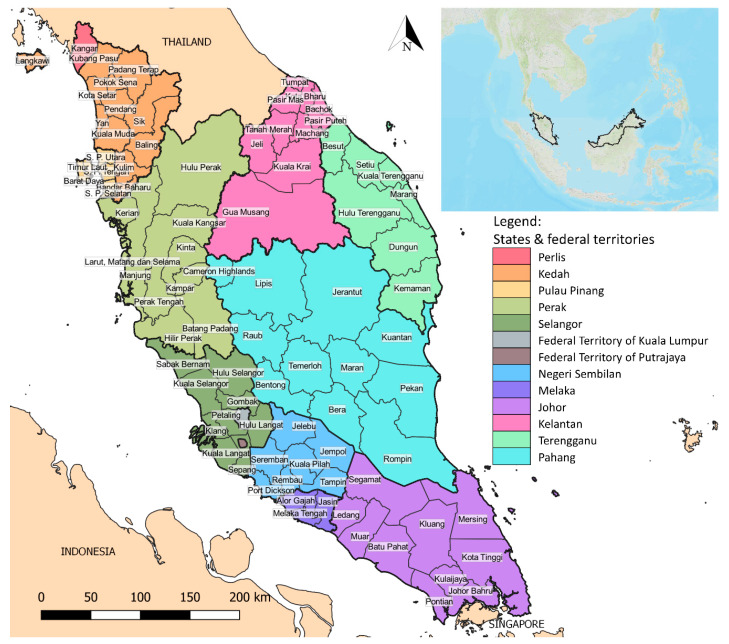
Peninsular Malaysia map showing states (divided into administrative levels of district) and federal territories.

Our study area primarily focused on Peninsular Malaysia which spreads from latitude 1°15′50.0″ N to 6°43′36″ N and from longitude 99°35′ E to 104°36′ E. Peninsular Malaysia covers a land area of 13.21 million hectares (Figure 1) [18]. Forested areas account for approximately 5.76 million hectares, which is equivalent to 43.6% of the land area of Peninsular Malaysia [18]. The climate is categorized as equatorial, typically hot and humid throughout the year. Rainfall pattern in Peninsular Malaysia is mainly influenced by the southwest monsoon season (from May to August) and northeast monsoon season (from November to February) [19]. The annual rainfall ranges from a maximum of 5000 to a minimum of 1750 mm. The mean daily temperature ranges from 25 to 28 °C.

Peninsular Malaysia is divided into 11 states (Perlis, Kedah, Pulau Pinang, Perak, Selangor, Negeri Sembilan, Melaka, Johor, Kelantan, Terengganu, and Pahang) and 2 federal territories (Kuala Lumpur and Putrajaya) (Figure 1). Approximately 24.7% of the population of Peninsular Malaysia reside in sub-urban and rural areas.

### 2.2. Data of Knowlesi Malaria in Peninsular Malaysia

Laboratory diagnosis of knowlesi malaria is conducted via microscopic examination or PCR-based approaches [20]. At present, District Health Offices notify the State Health Departments of the confirmed positive malaria cases, which will be further compiled by the Ministry of Health Malaysia. In this study, laboratory confirmed knowlesi malaria case data for the period 2011–2018 were provided by the Ministry of Health Malaysia. This dataset contains information on each confirmed case, including state, district, year, nationality, ethnicity, citizenship, occupation, age, gender, case classification, and date of onset. The population data of Peninsular Malaysia were obtained from the Department of Statistics Malaysia open-source platform [17,21]. The descriptive epidemiological analysis was conducted at the state level to show the demographic characteristics of indigenous *P. knowlesi* infection cases. An indigenous malaria case is a case contracted locally with no evidence of importation and no direct link to transmission from an imported case, whereas an imported malaria is a case in which the infection was acquired outside the area in which it was diagnosed [22]. The case data are available at both the state and district levels; however, we aggregated data based on district locations where the cases were reported for subsequent analysis.

This study was registered with the National Medical Research Register (NMRR-16-2109-32928), and ethical approval was obtained from the Malaysian Research Ethical Committee (MREC) (reference no. KKM/NIHSEC/P16-1782 (11)).

### 2.3. Spatial Analysis of Knowlesi Malaria in Peninsular Malaysia

Spatial analysis was conducted at the district level in Peninsular Malaysia for the period 2011–2018. The annual square root transformed incidence rate (IR) per million people for each district was calculated to deal with the highly skewness of raw incidence rate caused by outliers and deviation from normality of variance. We used the population estimates from the 2010 National Census data [17] and annual state population growth estimation from the Department of Statistics Malaysia [21] to calculate the annual mid-year population for each district from 2011 to 2018, assuming that the population growth rate of each district was the same as the state’s population growth. The calculated annual mid-year population was used to estimate annual IRs accurately.

Both Global and Local Moran’s I statistics were performed to identify the spatial autocorrelation and disease hotspots in the study area. First order queen contiguity spatial weight matrix was generated to define districts with shared borders and vertices as neighbors for subsequent analysis. Global Moran’s I test can be used to detect the spatial autocorrelation of *P. knowlesi* infections. It provides a continuous index value, which can be an indication of random distribution (value of zero or close to zero), clustered distribution (value close to +1.0), or dispersed but organized distribution (value close to −1.0) [23]. The equation for Global Moran’s I is as follows:

(1) $$I=\frac{n\sum_{i=1}^{n}\sum_{j=1}^{n}w_{ij}\left(x_{i}-\bar{x}\right)\left(x_{j}-\bar{x}\right)}{(\sum_{i=1}^{n}\sum_{j=1}^{n}w_{ij})\sum_{i=1}^{n}{\left(x_{i}-\bar{x}\right)}^{2}}$$

where *n* is the number of observations, $\bar{x}$ is the mean of the variable, *x_i_* is the variable value at a particular location, *x_j_* is the variable value at another location, *w_ij_* is a weight indexing location of *i* relative to *j*.

Local Moran’s I test involves the formation of Local Indicators of Spatial Association (LISA) statistics to detect statistically significant spatial clusters of a disease (hot spots and cold spots) as well as to identify outliers. The equation for Local Moran I statistics is as follows:

(2) $$I_{i}=z_{i}\;\underset{j}{\sum }w_{ij}z_{j\;},$$

with *z_i_* and *z_j_* are in deviations from the mean.

LISA values present four types of clusters: high-high, low-low, high-low, and low-high [24]. High-high clusters are associated with high IR areas (hot spots), whereas low-low clusters are associated with low IR areas (cold spots). High-low and low-high categories represent outliers. The statistic inferences of both tests were performed by Monte Carlo simulation with 99,999 permutations to generate the *p*-value. False Discovery Rate (FDR) correction was applied to reduce alpha risk (Type I error), and resulted in *p*-value = 0.008 being identified as the significance cut-off. Global and Local Moran’s I tests were performed using GeoDa 1.14 (University of Chicago, Chicago, IL, USA). Data visualization was performed with QGIS 3.6.3 (Open Source Geospatial Foundation, Beaverton, OR, USA) to demonstrate the spatial and temporal patterns of *P. knowlesi* in Peninsular Malaysia from 2011 to 2018.

### 2.4. Time Series Analysis of P. knowlesi Incidence in the Hotspot Region

Knowlesi malaria cases in all districts identified as hotspots were summed into a single dataset. The cases were aggregated by month to calculate the monthly IR from January 2012 to December 2018. Cases reported in 2011 were not included as the onset date information was not recorded. Trend and seasonality of the monthly IR was identified using stl() function in R version 3.5.3 (R Foundation for Statistical Computing, Vienna, Austria). Time series data of monthly IR was decomposed into trend and seasonality components using locally estimated scatterplot smoothing [25].

The dataset was assigned into a training period (January 2012 to December 2017) and a validation period (January 2018 to December 2018). The training period data were used to create and fit seasonal autoregressive integrated moving average (SARIMA) models whereas the validation period data were used to test the model forecasting performance. SARIMA forecasting procedure was conducted in R version 3.5.3 (R Foundation for Statistical Computing, Vienna, Austria) by utilizing fUnitRoots, forecast, and tseries packages. SARIMA model is generally denoted as SARIMA (p, d, q) (P, D, Q)_m_, which can be distinguished into non-seasonal components (p, d, and q) and seasonal components (P, D, Q, and m). In non-seasonal component notation, parameter p is the order of autoregressive (AR), d is the order of differencing, and q is the order of moving average (MA). Parameters P, D, and Q are the orders of AR, differencing, and MA, respectively, whereas m refers to the number of periods in each season, defined as 12 in this study.

The monthly knowlesi malaria incidence rates were corrected to achieve stationarity via differencing approach. Stationarity of the time series was tested using augmented Dickey–Fuller test and Kwiatkowski–Phillips–Schmidt–Shin (KPSS) unit root test. Order of differencing required was identified from the minimum number of differencing required to achieve stationarity. Orders of AR and MA terms were determined based on autocorrelation function (ACF) and partial autocorrelation function (PACF) plots of the differenced series (Figure A3). R functions, auto.arima() and arima(), were used for automatic parameters selection based on the Akaike information criterion (AIC), Bayesian information criterion (BIC), root mean square error (RMSE), and mean absolute percentage error (MAPE). Lower values of these diagnostic statistics indicate better model performances and prediction accuracy [26]. For diagnostic checking of the fitted models, the Ljung–Box test was used to evaluate the model residual patterns. The null hypothesis of the Ljung–Box test is that the residuals are random. Random residuals indicate the absence of significant temporal autocorrelation across multiple time lags, which could be displayed in ACF and PACF plots, and the model is not lack of fit. The models were excluded if the residuals showed sign of non-randomness (*p* < 0.05). The best-fitted model was used to forecast the IR of knowlesi malaria from January 2018 to December 2018. The model forecast with 80% and 95% prediction intervals was compared against the validation period dataset between January 2018 to December 2018.

## 3. Results

### 3.1. Demographic Characteristics of Knowlesi Malaria from 2011 to 2018

**Figure 2:**
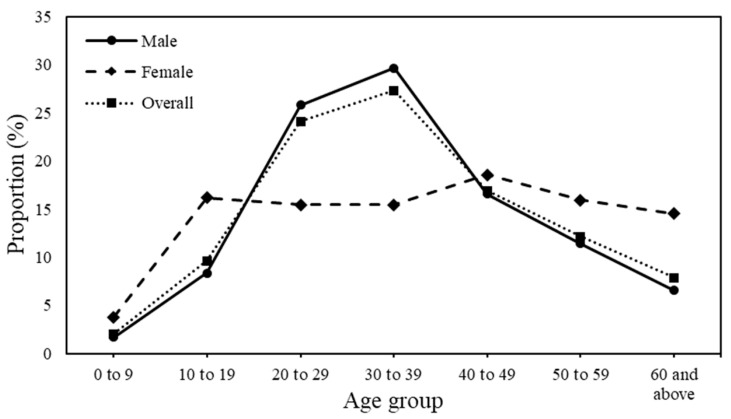
Proportion of indigenous knowlesi malaria cases by age and gender from 2011 to 2018.

**Figure A1:**
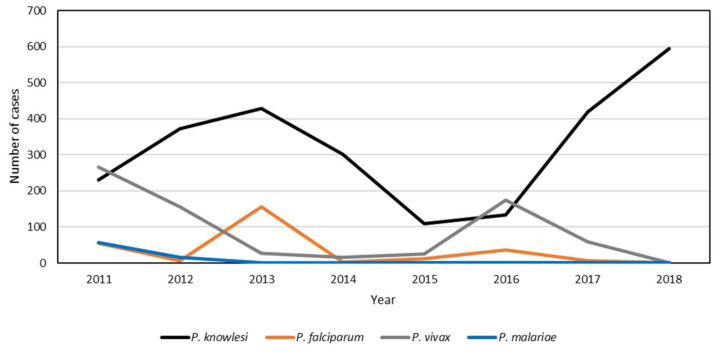
Number of indigenous malaria cases according to year and *Plasmodium* species.

**Figure A2:**
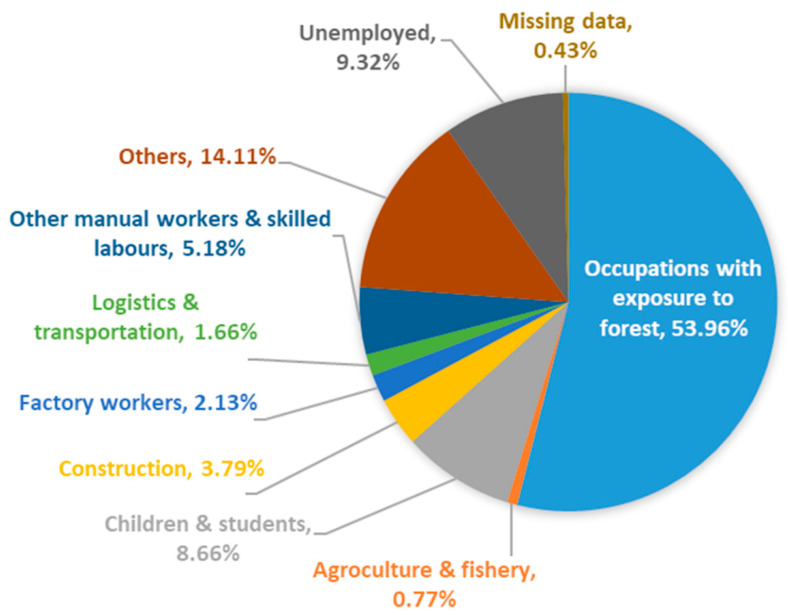
Types of occupation of indigenous knowlesi malaria patients. The proportion of occupations with exposure to forest is compared with other types of occupation.

**Figure A3:**
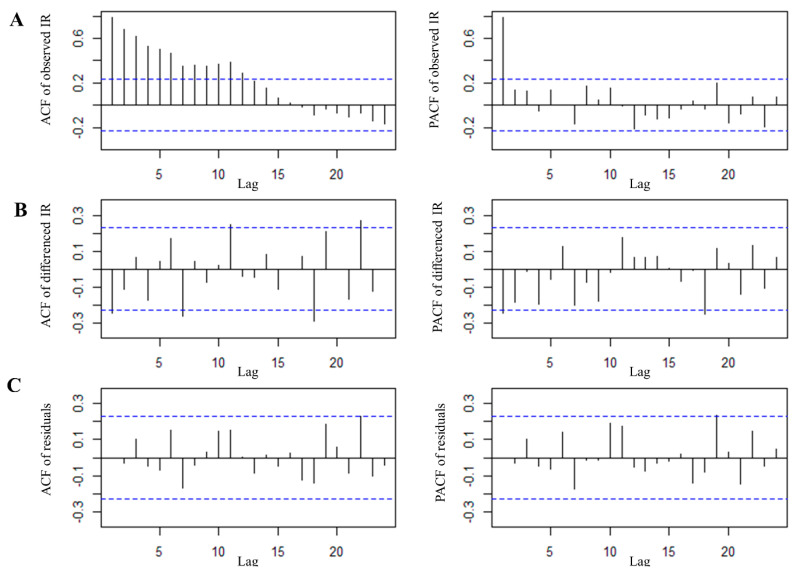
(**A**) Autocorrelation function (ACF) and partial autocorrelation function (PACF) plots of observed IR of knowlesi malaria from 2012 to 2017. (**B**) ACF and PACF plots of one-order non-seasonal and seasonal differenced knowlesi malaria IR series (d = 1 and D = 1). (**C**) ACF and PACF plots of fitted SARIMA (0,1,1)(2,1,0)_12_ model residuals. The dashed lines represent 95% confidence intervals.

The demographic characteristics of indigenous *P. knowlesi* cases in Peninsular Malaysia are shown in Table 1. From 2011 to 2018, 2587 indigenous *P. knowlesi* cases were reported in Peninsular Malaysia. The number of indigenous *P. knowlesi* cases was apparently higher than other indigenous human malaria cases (*P. malariae*, *P. falciparum*, and *P. vivax*) in most years except 2011 and 2016 (Figure A1). Although fewer females were infected with *P. knowlesi* as compared to males, female patients (median 39 years, interquartile range 23–52 years) were generally older than the males (median 34 years, interquartile range 25.5–45 years). Bimodal age pattern was displayed among female patients with the highest proportion among the age group of 40–49 years followed by 10–19 years, whereas the mode for male patients was the 30–39 years age group (Figure 2). Indigenous cases were more prevalent among the Malays (58.7%) than among other ethnicities. Foreigners accounted for 16.4% of the indigenous cases. Forest-related occupations accounted for 53.96% of the total indigenous cases. One-third of the total indigenous cases was reported among estate, farm, and plantation workers (Table 1 and Figure A2).

The age and gender distribution of indigenous *P. knowlesi* cases according to state are characterized in Table 2 and Table 3. *P. knowlesi* was significantly more prevalent in the population aged 20–29 years in Negeri Sembilan and Pahang, and aged 30–39 years in Johor, Kedah, Kelantan, Perak, Selangor, and Terengganu (Table 2). The incidence of *P. knowlesi* was generally higher among males than among females in most states (Table 3).

### 3.2. State-Level Trend of P. knowlesi Incidence in Peninsular Malaysia from 2011 to 2018

Between 2011 and 2018, a total of 2767 (include imported cases) *P. knowlesi* monoinfection cases were reported in Peninsular Malaysia. Of these cases, 812 (29.35%) were from Kelantan state, while 666 (24.07%) and 524 (18.94%) were from Pahang and Perak, respectively (Table 4).

**Figure 3:**
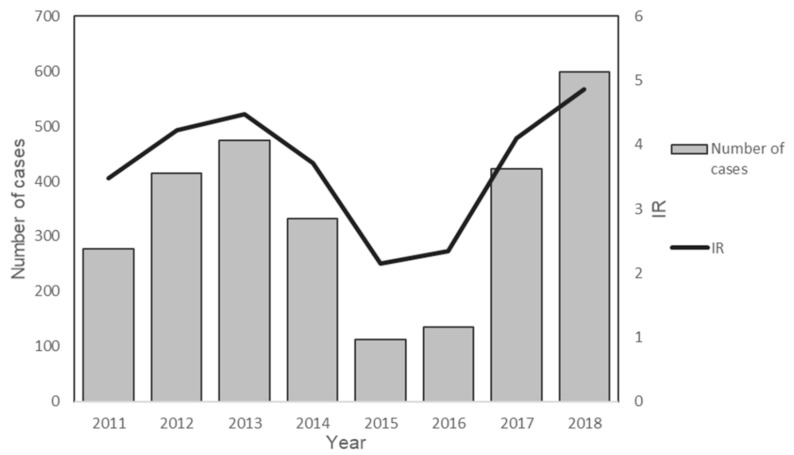
*P. knowlesi* monoinfection cases in Peninsular Malaysia from 2011 to 2018.

**Figure 4:**
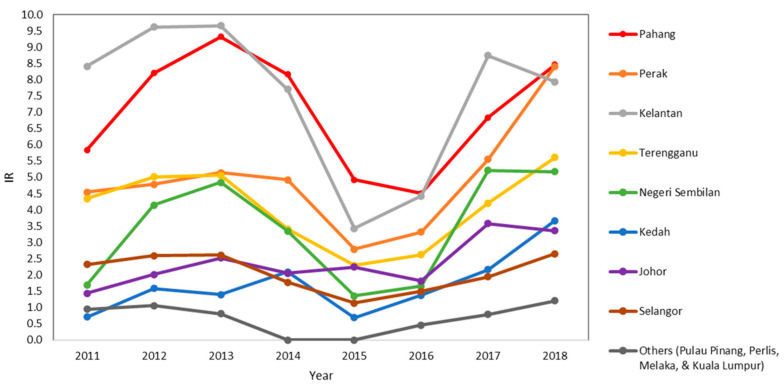
State-wide *P. knowlesi* incidence rate (IR) from 2011 to 2018.

The reported cases increased from 2011 to 2014, followed by a transient drop in 2015 and 2016 prior to gradual increase (Figure 3). In 2011, 277 cases were reported, and the number of cases recorded in 2018 was 598. A similar trend could be observed in the IR of five states: Kelantan, Pahang, Perak, Terengganu, and Negeri Sembilan. However, the IR in Kelantan and Negeri Sembilan dropped in 2018 as compared to 2017 (Figure 4). Federal Territory of Kuala Lumpur, Pulau Pinang, Melaka, and Perlis had an IR lower than 1.5 annually. No knowlesi malaria case was reported in the Federal Territory of Putrajaya throughout the study duration.

### 3.3. District-Level Spatial Analysis of P. knowlesi Infection

**Figure 5:**
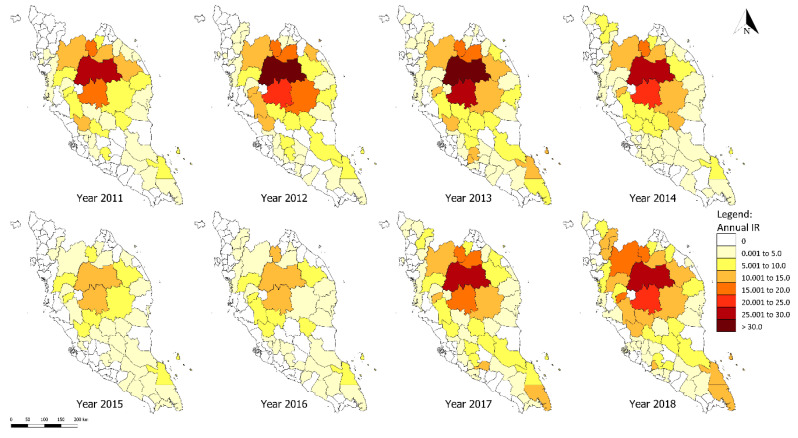
Spatial-temporal distribution of knowlesi malaria in Peninsular Malaysia (2011–2018).

The district-level *P. knowlesi* IR from 2011 to 2018 is illustrated in Figure 5. Overall, the spatial pattern of *P. knowlesi* transmission was higher in the central-northern region of Peninsular Malaysia. Gua Musang and Lipis districts consistently recorded higher IR annually throughout the study period. In 2015, there was a transient drop in the incidence of knowlesi malaria in almost all districts compared with that in previous years. Only two districts reported a high incidence in the same year. *P. knowlesi* incidence peaked in 2018 in 17 districts.

**Figure 6:**
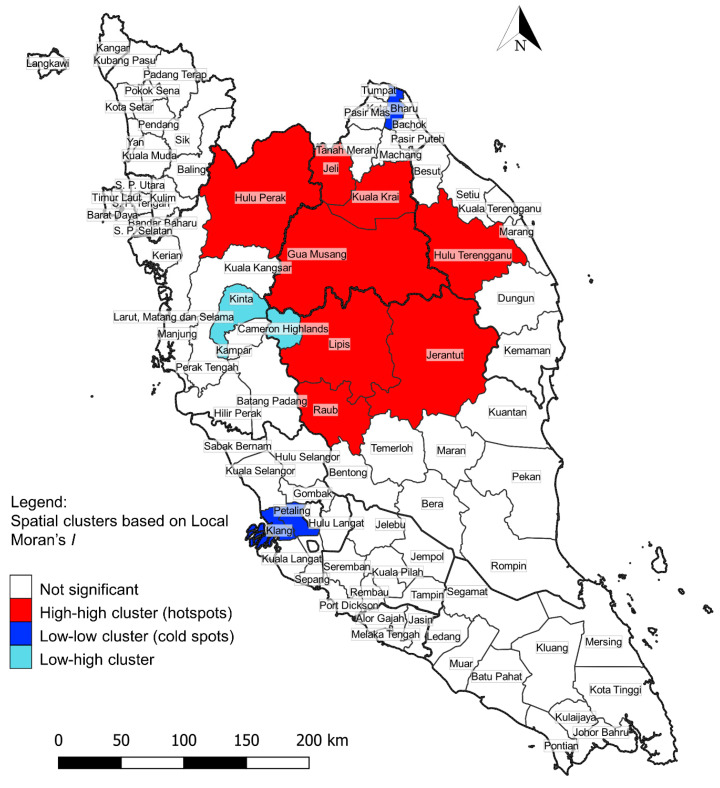
District-level hotspot and cold spot spatial clusters of knowlesi malaria (2011–2018).

The spatial autocorrelation of knowlesi malaria was evaluated using the 2011–2018 summarized cases. Global Moran’s I test revealed a significant and positive spatial autocorrelation in the study area (Global Moran’s I = 0.489, *p* < 0.001, Z = 7.02). This indicated that there was spatial dependence and clustering of *P. knowlesi* incidence in Peninsular Malaysia. Local Moran’s I test identified major hotspots (high–high spatial clusters) involving eight districts (Table 5 and Figure 6). Two cold spots (low–low spatial clusters) comprising three districts were identified near major cities. All hotspots were in regions with low population density (average of 34 people per square km), whereas cold spots comprised locations with high population density (average of 1305 people per square km). Low–high spatial clusters were identified in two districts. These two districts (Kinta and Cameron Highlands) had a low incidence of knowlesi malaria but were surrounded by districts with a high incidence of the disease.

### 3.4. Time Series Analysis of P. knowlesi Incidence in the Hotspot Region

**Figure 7:**
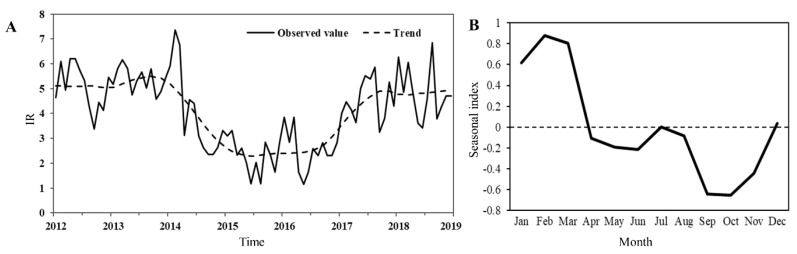
(**A**) Trend of monthly IR of knowlesi malaria in hotspots from 2012 to 2018. (**B**) Seasonality of knowlesi malaria incidence in hotspots.

Time series decomposition of the monthly IR of knowlesi malaria in hotspot region involving eight districts (Jeli, Kuala Krai, Jerantut, Lipis, Raub, Hulu Perak, Hulu Terengganu, and Gua Musang) is illustrated in Figure 7. The IR of knowlesi malaria in hotspots experienced a transient downward trend beginning from early 2014 prior to increase in 2017 (Figure 7A). This trend was similar to the general trend in Peninsular Malaysia. The seasonality of *P. knowlesi* incidence indicated the major peak between January and March (Figure 7B).

**Figure 8:**
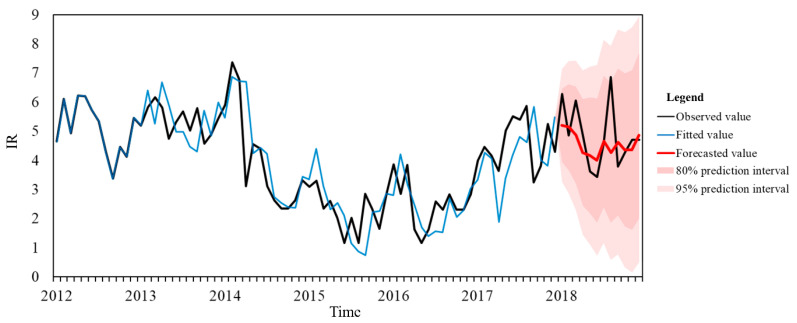
Time series plot of observed, fitted and forecasted values of knowlesi malaria IR using SARIMA (0,1,1)(2,1,0)_12_.

Five candidate SARIMA models with random pattern of the residuals were selected to compare the model performance. Overall, SARIMA (0,1,1)(2,1,0)_12_ was considered the best-fitted models of knowlesi malaria incidence in the hotspot region (AIC = 184.62, BIC = 192.93, RMSE = 0.90, MAPE = 18.51) (Table 6). This forecasting results demonstrated that the model captured the temporal patterns in 2018 but missed the peak in August (Figure 8).

## 4. Discussion

This study demonstrated the fundamental epidemiology of *P. knowlesi* infection in Western Malaysia. *P. knowlesi* infections were distributed across all age groups with a higher prevalence among adults aged 20–39 years, specifically in men, who are likely to be more active outdoors and have greater forest exposure owing to job requirements [27]. A study in Borneo showed that men accounted for 85% of PCR-confirmed knowlesi malaria cases [14]. This proportion of male *P. knowlesi* patients is similar to that reported in nine states in Peninsular Malaysia (79.92%–93.33%). A lower incidence of *P. knowlesi* cases was observed among children and the elderly, which could be associated with limited outdoor activities and lower risk of getting bit by infected *Anopheles* mosquitoes.

Individuals infected with *P. knowlesi* were involved in various types of occupation. However, most (53.96%) had occupations related to agriculture and the forest, and this phenomenon has been shown in a previous study [15]. Nonetheless, types of occupation alone may not provide a true reflection of the risk of *P. knowlesi* infection. Recreational forest activities such as bird watching, hiking, and camping may also expose an individual to infective mosquito bites. Rural and suburban settlements located close to the forest and in forested areas mostly comprise the Malay community, which suggests a greater burden of *P. knowlesi* infection among Malays than among other ethnic groups.

In this study, Kelantan, Pahang, and Perak had consistently recorded high burden of knowlesi malaria. These states have the largest settlement of aborigine communities [28]. Aborigines made up one-tenth of the total indigenous knowlesi malaria cases. Prior to our study, human malaria parasitic infections had been frequently reported among the aborigine population in Peninsular Malaysia [29,30,31]. Moreover, researchers detected the presence of submicroscopic *P. knowlesi* infections among asymptomatic individuals within these communities [32]. Aborigine communities are considered at a high-risk of exposure to malaria. This is because their settlements are located in the forests and forest fringes, and many are still dependent on forest resources for subsistence [33,34]. Nevertheless, logistical hurdles and the presence of submicroscopic incidence within these communities have challenged the current microscopic and PCR-based diagnostic approaches. These justify the necessity for development of portable and highly sensitive *P. knowlesi* specific rapid diagnostic tests.

Spatial analysis indicated a high IR of *P. knowlesi* concentrated in the central-northern region of Peninsular Malaysia. Within this region, Gua Musang and Lipis districts reported the highest IR when compared with other districts. They are neighboring districts and most infected patients worked in the agricultural sector, implying frequent exposure to the forest, forest-edge, and plantation setting. Thus, this increased probability of contact with *Anopheles* mosquitoes as well as macaque populations. Prior to 2015, many agricultural, logging, and quarrying activities occurred in the Gua Musang district [35]. These activities included exploitation of secondary forests and permanent forest reserves, which potentially led to the spill over of the macaque population to human settlements. A 2011 census on long-tailed macaque density covering all Peninsular Malaysia states estimated that the macaque population was the highest in agricultural areas, followed by oil palm plantations and urban areas, possibly due to abundant food resources for macaques [36]. The mosquito vectors may have followed their macaque hosts and adapted to these habitats [37]. To date, point prevalence of *P. knowlesi* has been established for wild macaque population in Johor (3%), Perak (4%), Pahang (26%) [38], and Selangor (30%) [39]. Additionally, incriminated vectors for *P. knowlesi* including *Anopheles cracens* and *Anopheles introlatus* were found in Pahang and Selangor, respectively, with a *P. knowlesi* positive rate of less than 2% [9,40]. Natural simian malaria infections in humans tend to occur when humans break the normal mosquito–macaque circulation chain in the forested area [41]. Current knowledge on *P. knowlesi* prevalence among macaques and *Anopheles* mosquitoes is scarce, and there is a need to increase surveillance coverage for providing improved information on parasite prevalence in whole Peninsular Malaysia.

A decline in *P. knowlesi* incidence was observed in 2015 and 2016, whereby the number of knowlesi malaria cases reported in Peninsular Malaysia dropped below 150 annually. Possible explanations for this change include a transient reduction in case detection activities, reduction in vector density, reduction in macaque population, or reduction in human-mosquito contacts. Malaria elimination efforts were intensified to reduce the population of vectors responsible for human malaria such as *P. malariae*, *P. vivax*, and *P. falciparum*, and these activities could have temporarily reduced the *P. knowlesi* vector population as well. These measures include the distribution of ITNs, larviciding, residual spraying, and repellents to high-risk groups to reduce the malaria vector population and human-mosquito contacts. A successful malaria health policy could lead to a reduction in malaria cases [42]. In addition, the climate change phenomenon, which led to an anomalous rainfall pattern and strong drought in Southeast Asia in 2015, accompanied by severe haze episodes, could have contributed to changes in vector density and reduced malaria transmission [14,43,44]. This highlights the need to assess the effect of extreme climatic variability on changes in *P. knowlesi* transmission [42].

A significant rebound in *P. knowlesi* transmission was observed after 2016. This may be due to drastic changes in the transmission dynamics of the parasite between humans, macaques, and vectors caused by agricultural expansion and forest exploration. According to the Forestry Department of Peninsular Malaysia statistical report, approximately one million hectares of total forested areas have been lost from 2016 to 2018, and the permanent reserved forest area has decreased from 4.92 million hectares in 2016 to 4.80 million hectares in 2018 [18]. Peninsular Malaysia has experienced large-scale deforestation due to intensified agricultural activities such as harvesting oil palm and rubber, timber production, and rapid urban expansion since the 1970s [45]. Establishment of crop plantations increases the vectors’ natural breeding site and human exposure to these vectors’ breeding sites [46]. In contrast to *P. falciparum* or *P. vivax* transmission, people infected by *P. knowlesi* are usually exposed to agricultural settings or forests instead of contracting the infection at home or surrounding areas owing to the exophagic nature of the vectors [37]. In Sabah, a higher *P. knowlesi* transmission was observed in large intact forest patches within 5 km of households, pulpwood plantations within 3 km of households, and oil palm plantations with fragmented landscapes [15]. Malaria elimination strategies such as ITN and residual spraying applied within the vicinity of houses have proven to be effective in interrupting human malaria transmission but less protective against simian malaria vectors that feed on its host predominantly in the forest. However, personal insecticide usage has been proven to reduce the risk of exposure to *P. knowlesi* [15]. Although efforts have been made to distribute repellents to agricultural workers, their active participation in using these repellents routinely at work is required. Furthermore, loss of cross-species immunity may have contributed to the surge of knowlesi malaria incidence especially after 2016 following a decrease in human malaria parasites incidence, and this was similarly postulated in a previous study [14]. For instance, early studies presented that individuals with history of *P. vivax* infection were more resistant to *P. knowlesi* infection than those without *P. vivax* infection history, and recently, *P. knowlesi* erythrocyte invasion inhibition by *P. vivax* antibodies was reported [47,48,49].

As both Peninsular Malaysia and Malaysian Borneo share similar geographical and cultural characteristics to a certain extent, there are other comparative points from previous studies that can reflect the transmission of *P. knowlesi* infections in Peninsular Malaysia. Both Peninsular Malaysia and Malaysian Borneo experienced an increase in *P. knowlesi* infections despite a dramatic decline in other human malaria parasite infections [16]. In Sabah, a higher rainfall was associated with an increase in knowlesi malaria cases after three months [14,50]. An increase in rainfall causes the formation of water pockets, which are ideal for mosquito breeding. Nevertheless, a combination of environmental factors, including temperature, rainfall, humidity, and land use, could serve as predictors of disease transmission. In addition, community-level surveillance using serological markers demonstrated that *P. knowlesi* exposure was positively associated with age, the male sex, forest activity, and contact with macaques but negatively with personal insecticide practice and residing at a higher altitude [15,51]. Similar investigations should be conducted to understand the environmental and exposure risks based on these factors, particularly in Peninsular Malaysia.

In this study, some districts in Perak, Kelantan, Pahang, and Terengganu states were found to have *P. knowlesi* hotspots. These findings can guide malaria control programs in strategizing effective malaria intervention, specifically in these districts. Moreover, the monitoring of districts clustered as hotspots is crucial because disease transmission may spill over to their neighbors owing to cross-district movement of macaque populations with potential carriage of malaria parasites or long-distance spread of infective vectors. Studies in Africa suggested that some species of malaria vectors can fly over hundreds of kilometers [52,53]. To date, the dispersal behaviors of malaria vector species in Malaysia are not fully understood, and this has certainly revealed a knowledge gap for further studies to determine the connectivity between bionomics of vectors and malaria incidence.

SARIMA models were widely applied by researchers to predict burden of infectious diseases, including malaria [54,55,56], dengue [57], influenza [58,59], and mumps [60]. SARIMA (0,1,1)(2,1,0)_12_ were found to be the best suited models for future short time frame prediction of knowlesi malaria incidence in major malaria hotspots in Peninsular Malaysia. Although all the observed IR from January 2018 to December 2018 can be predicted within 80% prediction interval, the model missed the peak in August, which indicated that some latent parameters should be included in the future to improve prediction accuracy. These parameters could be associated with vectors or reservoir hosts abundance, environmental factors, and public health interventions. Comparatively, researchers in Sarawak developed a non-seasonal autoregressive model to predict statewide increasing trend of *P. knowlesi* transmission up to 2040; however, such long-term forecasting might encounter various uncertainty issues [61]. The short-term forecasting model developed in this study might be more feasible for control strategy modification.

This is the first study that utilized spatial data analysis to generate the spatial distribution of knowlesi malaria at the district level covering the entire Peninsular Malaysia. Nonetheless, this study has some limitations. First, the study was descriptive in nature, with no control group for risk factor analysis. Second, we did not include environmental parameters in the analysis, which are important to better describe the transmission dynamics of *P. knowlesi* in the study area. Third, data related to the macaque reservoir host were not included in this study because there was a lack of comprehensive surveillance data for macaques infected with *P. knowlesi* from 2011 to 2018. Further studies could be conducted by considering the impact of environmental variations on the transmission of knowlesi malaria. Factors such as rainfall, relative humidity, temperature, water bodies, and normalized difference vegetation index can be used to spatially assess the malaria risk factors. The availability of macaque host data could assist in understanding the role of the macaque population, particularly those carriers for *P. knowlesi*, in disease transmission to humans. Although this study does not involve macaque data, its output could suggest focal areas for surveillance of *P. knowlesi* within the macaque population in future research. While Malaysia is moving forward to end the transmission of human malaria, the emergence of *P. knowlesi* could become the next challenge for malaria elimination programs. Advancement in our knowledge about the ecology of *P. knowlesi* could help policy makers develop effective *P. knowlesi*-specific control strategies in Peninsular Malaysia.

## 5. Conclusions

This study highlighted the spatial distribution of *P. knowlesi* at the district level from 2011 to 2018 in Peninsular Malaysia. Coupled with the temporal forecasting method using SARIMA, the findings from this study could assist malaria elimination programs in targeting *P. knowlesi* hotspots in Peninsular Malaysia for programmatic intervention.

## Figures and Tables

**Table 1:** Demographic characteristics of indigenous *P. knowlesi* cases in Peninsular Malaysia from 2011 to 2018.

Variable	N (%)
Age group	
0 to 9	53 (2.0)
10 to 19	249 (9.6)
20 to 29	624 (24.1)
30 to 39	707 (27.3)
40 to 49	437 (16.9)
50 to 59	314 (12.1)
60 and above	203 (7.8)
Gender	
Male	2167 (83.8)
Female	420 (16.2)
Ethnicity	
Chinese	145 (5.6)
Indian	44 (1.7)
Malay	1518 (58.7)
Orang Asli (aborigine community)	372 (14.4)
Sabahan	27 (1.0)
Sarawakian	34 (1.3)
Others	429 (16.6)
No data	18 (0.7)
Occupation class	
Agroculture and fishery	20 (0.8)
Army and police *	138 (5.3)
Children and students	224 (8.7)
Construction	98 (3.8)
Ecotourism, forest, and wildlife management *	39 (1.5)
Estate, farm, and plantation workers *	897 (34.7)
Factory workers	55 (2.1)
Forest resource gatherers *	82 (3.2)
Logging *	121 (4.7)
Logistics and transportation	43 (1.7)
Mining *	51 (2.0)
Other manual workers and skilled labors	134 (5.2)
Village labors *	68 (2.6)
Others	365 (14.1)
Unemployed	241 (9.3)
No data	11 (0.4)
Citizenship	
Locals	2161 (83.5)
Foreigners	423 (16.4)
No data	3 (0.1)

* Occupations with exposure to the forest.

**Table 2:** Age-based distribution of indigenous knowlesi malaria according to states from 2011 to 2018.

Age Group	Number of Indigenous Knowlesi Malaria Monoinfection Cases According to States (%)											
	Johor	Kedah	Kelantan	Melaka	Negeri Sembilan	Pahang	Perak	Perlis	Pulau Pinang	Selangor	Terengganu	Kuala Lumpur
0 to 9	0 (0.0)	1 (1.7)	18 (2.7)	0 (0.0)	3 (2.5)	16 (2.4)	14 (2.7)	0 (0.0)	0 (0.0)	1 (0.5)	0 (0.0)	0 (0.0)
10 to 19	4 (2.4)	0 (0.0)	83 (12.2)	1 (14.3)	6 (5.1)	59 (8.9)	60 (11.6)	0 (0.0)	1 (16.7)	24 (11.4)	11 (6.9)	0 (0.0)
20 to 29	47 (28.5)	8 (13.3)	165 (24.3)	2 (28.6)	39 (33.1)	167 (25.2)	107 (20.7)	0 (0.0)	0 (0.0)	55 (26.2)	34 (21.3)	0 (0.0)
30 to 39	58 (35.2)	24 (40.0)	176 (26.0)	1 (14.3)	24 (20.3)	163 (24.6)	137 (26.4)	1 (100.0)	2 (33.3)	66 (31.4)	55 (34.4)	0 (0.0)
40 to 49	33 (20.00)	12 (20.0)	114 (16.8)	1 (14.3)	19 (16.1)	108 (16.3)	93 (18.0)	0 (0.0)	0 (0.0)	29 (13.8)	27 (16.9)	1 (100.0)
50 to 59	17 (10.3)	12 (20.0)	75 (11.1)	1 (14.3)	17 (14.4)	86 (13.0)	66 (12.7)	0 (0.0)	1 (16.7)	20 (9.5)	19 (11.9)	0 (0.0)
60 and above	6 (3.6)	3 (5.0)	47 (6.9)	1 (14.3)	10 (8.5)	64 (9.7)	41 (7.9)	0 (0.0)	2 (33.3)	15 (7.1)	14 (8.8)	0 (0.0)
Total	165 (100.0)	60 (100.0)	678 (100.0)	7 (100.0)	118 (100.0)	663 (100.0)	518 (100.0)	1 (100.0)	6 (100.0)	210 (100.0)	160 (100.0)	1 (100.0)

**Table 3:** Gender-based distribution of indigenous knowlesi malaria cases according to states from 2011 to 2018.

State	Number of Indigenous Knowlesi Malaria Cases According to Gender (%)		Total (%)
	Male	Female	
Johor	157 (93.15)	8 (4.85)	165 (100.00)
Kedah	56 (93.33)	4 (6.67)	60 (100.00)
Kelantan	576 (84.96)	102 (15.04)	678 (100.00)
Melaka	6 (85.71)	1 (14.29)	7 (100.00)
Negeri Sembilan	98 (83.05)	20 (16.95)	118 (100.00)
Pahang	546 (82.35)	117 (17.65)	663 (100.00)
Perak	414 (79.92)	104 (20.08)	518 (100.00)
Perlis	1 (100.00)	0 (0.00)	1 (100.00)
Pulau Pinang	4 (66.66)	2 (33.33)	6 (100.00)
Selangor	181 (86.19)	29 (13.81)	210 (100.00)
Terengganu	128 (80.00)	32 (20.00)	160 (100.00)
Kuala Lumpur	0 (0.00)	1 (100.00)	1 (100.00)

**Table 4:** Number of cases of knowlesi malaria in Peninsular Malaysia and by states from 2011 to 2018.

States	Annual Number of Knowlesi Malaria Cases								Total	%
	2011	2012	2013	2014	2015	2016	2017	2018		
Total	277	414	474	332	113	136	423	598	2767	100
Johor	7	14	22	15	18	12	47	42	177	6.40
Kedah	1	5	4	9	1	4	10	29	63	2.28
Kelantan	111	148	152	99	20	34	135	113	812	29.35
Melaka	0	1	1	0	0	0	2	4	8	0.29
Negeri Sembilan	3	18	25	12	2	3	30	30	123	4.45
Pahang	52	104	136	106	39	33	77	119	666	24.07
Perak	49	55	64	59	19	27	76	175	524	18.94
Perlis	0	0	0	0	0	0	0	1	1	0.04
Pulau Pinang	3	4	2	0	0	0	1	1	11	0.40
Selangor	30	38	40	19	8	14	24	45	218	7.88
Terengganu	20	27	28	13	6	8	21	38	161	5.82
Federal Territory of Kuala Lumpur	1	0	0	0	0	1	0	1	3	0.11
Federal Territory of Putrajaya	0	0	0	0	0	0	0	0	0	0.00

**Table 5:** Statistically significant district-level spatial clusters of knowlesi malaria based on IR, 2011–2018.

Cluster Type	District	Number of Cases	IR	LISA Index	*p*-Value
High-high	Jeli	95	46.24	5.88	<0.001
	Kuala Krai	167	37.35	2.92	0.001
	Jerantut	101	32.28	2.16	<0.001
	Lipis	329	58.70	5.97	<0.001
	Raub	66	25.49	1.51	0.003
	Hulu Perak	98	32.23	2.14	0.003
	Hulu Terengganu	68	29.42	1.36	0.008
	Gua Musang	506	71.68	6.88	<0.001
Low-low	Kota Bharu	1	1.36	0.48	0.006
	Klang	1	1.03	0.54	0.008
	Petaling	3	1.22	0.44	0.008
Low-high	Cameron Highlands	2	6.99	−0.58	<0.001
	Kinta	48	7.78	−0.25	0.006

**Table 6:** Comparison of candidate SARIMA models based on penalized criteria and accuracy measurement.

Models	AIC	BIC	RMSE	MAPE	Ljung–Box Test ^#^
SARIMA (0,1,0)(2,1,0)_12_	190.37	196.60	0.93	18.92	0.08
SARIMA (0,1,1)(2,1,0)_12_	184.62	192.93	0.90	18.51	0.50
SARIMA (0,1,1)(2,1,1)_12_	186.65	197.03	0.87	18.93	0.19
SARIMA (1,1,0)(2,1,0)_12_	186.14	194.45	0.89	18.52	0.36
SARIMA (2,1,0)(2,1,0)_12_	185.91	196.30	0.89	18.17	0.38

AIC: Akaike Information Criterion, BIC: Bayesian Information Criterion, RMSE: root mean square error, MAPE: mean absolute percentage error, df = degree of freedom; #: *p*-values of Ljung–Box test.
